# Editorial: Structural and Functional Organisation of the Prefrontal Cortex

**DOI:** 10.3389/fnsys.2016.00087

**Published:** 2016-11-09

**Authors:** Chris J. Tinsley

**Affiliations:** School of Science and Technology, Nottingham Trent UniversityNottingham, UK

**Keywords:** prefrontal cortex, structure, cortical function, cognition, connectivity

Prefrontal cortex (PFC) has long been known to be a region of the brain crucially important for cognitive processes. Major progress has been made in understanding the functions of this part of the brain however when it comes to brain organization there are still unanswered questions. In particular, if we compare known prefrontal cortex organization to that seen in sensory cortices, it is not currently clear whether PFC organization is essentially the same or different. This research topic focuses on the structural and functional organization of the prefrontal cortex in rodents and primates and makes a contribution to current research and debate within this field of neuroscience.

In the articles by Bedwell et al.; Bedwell et al., neuroanatomical tracers were employed to investigate PFC cortical connections. These studies illuminate a connectional feature common to PFC and other cortical regions: topological/topographical organization of connections. In addition, these studies provided evidence at a fine microscopic resolution for both reciprocal and non-reciprocal PFC connections. The finding of fine-scale non-reciprocity is unexpected, especially when one considers that other cortical regions (such as sensory cortices) are well-known for their detailed reciprocity. The study by Nguyen et al. uses electrophysiological approaches to explore important functional questions about the anatomical connections between PFC and primary visual cortex. Such links may be important for mediating attentional and other top-down signals. The experiments provided evidence for a functional link between infralimbic and prelimbic cortex and V1, but they found that this link was not mediated strongly via basalo-cortical cholinergic projections. Another feature of anatomical organization is the columnar organization present in PFC and in other regions of the mammalian cerebral cortex. Several previous anatomical studies have provided evidence for columnar or modular organization within PFC, in this topic Opris et al. investigated the functional aspects of this organization during exposure to cocaine. The results help to show how cocaine interrupts complex cognitive processing within columns and layers of the monkey prefrontal cortex.

Prefrontal cortex is perhaps best well-known for its role in cognitive function. The classic experiments of Funahashi and colleagues in the late 1980s and early 1990s (Funahashi et al., 1989, 1993) helped to define a role for the primate dorsolateral prefrontal cortex in spatial working memory processes. These studies were ground-breaking in terms of their contribution to our understanding of memory processes but also to the organization of neuronal responses within the PFC. In the review paper by Funahashi in this research topic, the interpretation of delay period activity in relation to visual memory tasks in primates is explored. The article presents previous and historical findings on the subject and discusses different interpretations of these important results. Another important function ascribed to the PFC is fear processing. PFC is known to have strong anatomical links with the amygdala, which is an important structure for mediating fear responses. This research topic features two articles which indicate that PFC plays a role in the processing of fear-related stimuli (Sharpe and Kilcross; Shiba et al.). Sharpe and Killcross investigated how cues modulate conditioned fear using inactivation of the rat prelimbic cortex. The authors found evidence for a prelimbic cortex role in the processing of these behavioral cues so that responses can be modulated accordingly. Shiba et al. explored how PFC lesions alter innate fear in the primate brain. Here the authors found that lesions to either ventrolateral PFC and orbitofrontal PFC caused heightened fear in response to predatory stimuli.

Some of the most sophisticated brain functions are now being allocated to the PFC. In a review of human neuroimaging studies Jeon discusses the progress made in understanding hierarchical processing within Prefrontal cortex. This review argues for importance of a small region of PFC in this high level brain function, namely Brodmann's area 44.

Another crucial cognitive function is attention and there is substantial and increasing evidence for the role of PFC in attention. In the imaging case study by Japee et al. the role of the right middle frontal gyrus (MFG) in reorienting attention was investigated. In this study a patient with MFG resection was compared to healthy volunteers and the results showed that, in certain tasks, the patient found it difficult in switching to top-down attentional control. In the review article by Cassaday et al., PFC studies involving attention are discussed in relation to the methodology employed and the results from different regions of PFC. This article covers studies of both rodent and primate PFC and outlines the evidence for distinct PFC regions having a role in both attention and executive processes. Remaining on the subject of methodology is the field of optogenetics which has become an increasingly important approach used in the arena of cognitive neuroscience. In the review article by Riga et al., the subject of how optogenetic tools have been used to study the prefrontal cortex in animal models is explored and discussed. The article highlights how these tools have been used to effectively study the functional circuitry of PFC in rodents. Optogenetics continues to be developed rapidly and successfully combined with existing techniques, such as fMRI (Liang et al., 2015).

This research topic provides a snapshot of current PFC research and highlights some of the progress made in understanding PFC anatomy and function. Many of these studies also point to the important role of PFC microcircuitry in cognitive processing. Clearly there are still important and fundamental gaps in our understanding of PFC structure and function. There are many similarities between the anatomical organization of PFC and other cortical regions yet functionally PFC may be unique, possibly having a multifunctional role which is difficult to define (Duncan, 2001). We still do not understand in detail how information flows or mediates cognitive processing in this crucial region however the new molecular biology techniques are helping scientists to address this question.

## Author contributions

The author confirms being the sole contributor of this work and approved it for publication.

### Conflict of interest statement

The author declares that the research was conducted in the absence of any commercial or financial relationships that could be construed as a potential conflict of interest.
